# Massive Cutaneous Xanthomas and Critical Triple-Vessel Coronary Artery Disease From Familial Hypercholesterolemia in a 40-Year-Old Woman

**DOI:** 10.7759/cureus.92277

**Published:** 2025-09-14

**Authors:** Jai Bharat Sharma, Kunal Mahajan, Surender Himral, Shivali Sandal, Tanuja Vats

**Affiliations:** 1 Department of Cardiology, Himachal Heart Institute, Mandi, IND; 2 Department of Critical Care Medicine, Himachal Heart Institute, Mandi, IND; 3 Department of Health and Family Welfare, Health Services Himachal Pradesh, Himachal Pradesh Government, Mandi, IND

**Keywords:** coronary artery disease (cad), familial hypercholesterolemia, premature atherosclerosis, xanthomas, young

## Abstract

Familial hypercholesterolemia is a monogenic inherited disorder of lipoprotein metabolism that often remains undiagnosed, primarily due to a lack of awareness rather than its rarity. We report the case of a 40-year-old unmarried woman who presented with worsening symptoms of exertional angina over six months. Physical examination revealed large xanthomas involving the dorsal aspect of both hands and feet and the extensor aspect of both knees and elbows and buttocks and a necklace-like lesion over the neck and upper chest, which had developed since her mid-20s. Her past and family history were suggestive, as her father had succumbed to myocardial infarction at 43 years of age. Laboratory evaluation demonstrated severe hypercholesterolemia with a total cholesterol of 485 mg/dl and low-density lipoprotein cholesterol of 398 mg/dl, with normal triglycerides and high-density lipoprotein cholesterol. Other systemic and metabolic parameters were within normal limits. Cardiovascular evaluation detected a soft systolic murmur, mild calcific aortic valve sclerosis, and mild aortic regurgitation, and duplex ultrasound revealed significant carotid artery stenoses. Coronary angiography demonstrated marked aortic root calcification and critical triple-vessel coronary artery disease. The diagnosis of heterozygous familial hypercholesterolemia was established clinically; genetic analysis could not be performed. The patient was advised to undergo coronary artery bypass grafting and started on high-dose rosuvastatin and ezetimibe, but could not afford proprotein convertase subtilisin/kexin type 9 (PCSK9) inhibitors. This case highlights the challenges in the early diagnosis and effective management of familial hypercholesterolemia, particularly in resource-constrained settings, underscoring the need for physician awareness and family-based screening to prevent premature atherosclerotic cardiovascular disease.

## Introduction

Familial hypercholesterolemia (FH) is now recognized as one of the most common inherited lipid disorders worldwide and a leading cause of premature atherosclerotic cardiovascular disease (ASCVD), particularly in South Asian populations [1,2]. Recent global and regional consensus statements estimate that heterozygous FH affects approximately one in 250-300 individuals, which translates to over 35 million people globally, yet the majority remain undiagnosed or undertreated [3,4]. In India, population studies have revealed both a higher prevalence of premature coronary artery disease (CAD) and a significant burden of undetected FH, highlighting the urgency for improved awareness and systematic screening [1-3]. The International Atherosclerosis Society and 2023 European Atherosclerosis Society guidelines underscore that untreated FH results in lifelong markedly elevated low-density lipoprotein cholesterol (LDL-C) levels, conferring a three- to fivefold increase in lifetime risk of CAD and a substantially heightened risk for early myocardial infarction [4,5].

Management of FH has advanced considerably in recent years. Early identification through clinical and genetic screening, initiation of high-intensity statins, combination therapy with ezetimibe, and, when needed, proprotein convertase subtilisin/kexin type 9 (PCSK9) inhibitors or lipoprotein apheresis now constitute the cornerstone of care [4,5]. Despite robust evidence for lowering LDL-C, uptake of advanced therapeutics remains challenging in resource-limited settings [1]. National and international guidelines also promote the cascade screening of close relatives and advocate multidisciplinary approaches to reduce the overall cardiovascular risk burden [4,5]. In the context of CAD, treatment paradigms have evolved alongside growing evidence. While beta-blockers have traditionally been regarded as a standard cornerstone therapy following myocardial infarction, recent major clinical studies and meta-analyses now suggest that their long-term prognostic benefit is largely confined to specific subgroups, particularly those with reduced left ventricular ejection fraction (LVEF) or overt clinical heart failure [6]. For patients such as ours, who present with premature and severe CAD due to FH yet have preserved LVEF, current guidelines recommend beta-blockers primarily for acute ischemic control, for arrhythmia management, and in settings of ventricular dysfunction, but not necessarily for long-term secondary prevention if LVEF is normal. This more nuanced, risk-adapted approach underscores the importance of individualizing therapy in complex cases of FH where traditional and novel therapies intersect [7].

In summary, FH represents a major, underrecognized driver of premature CAD in India and worldwide. Early detection, aggressive lipid-lowering therapy, tailored guideline-directed cardiovascular management, and a focus on family screening are critical for changing the natural history of this devastating genetic condition.

## Case presentation

**Figure 1:**
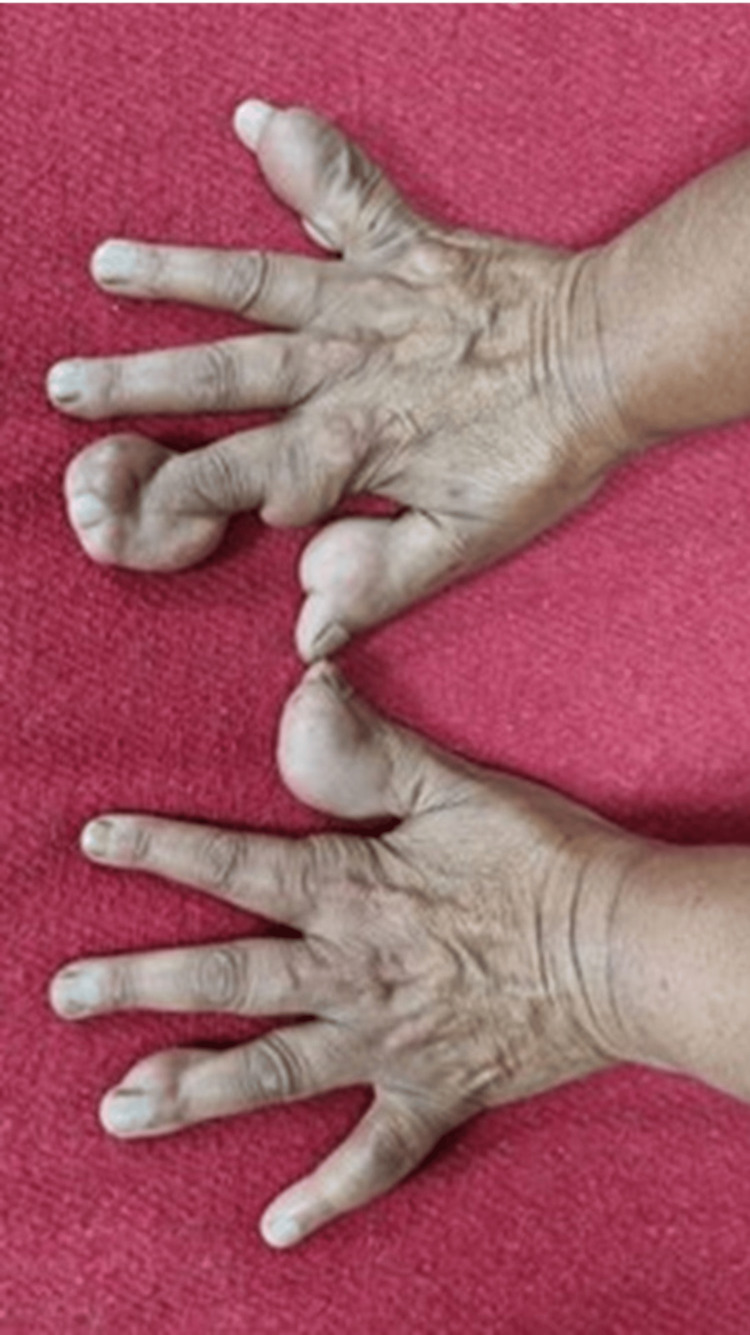
Xanthomas over the dorsal aspect of both hands

**Figure 2:**
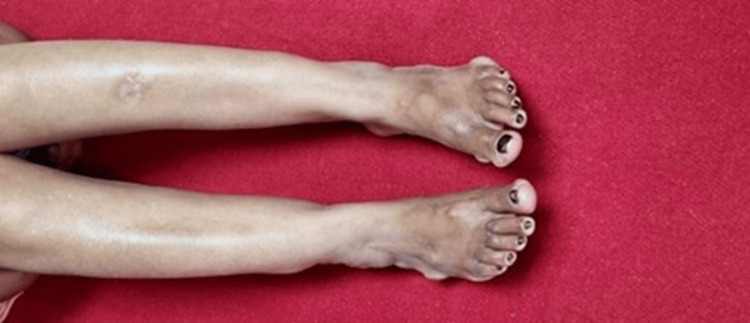
Xanthomas over both feet

**Figure 3:**
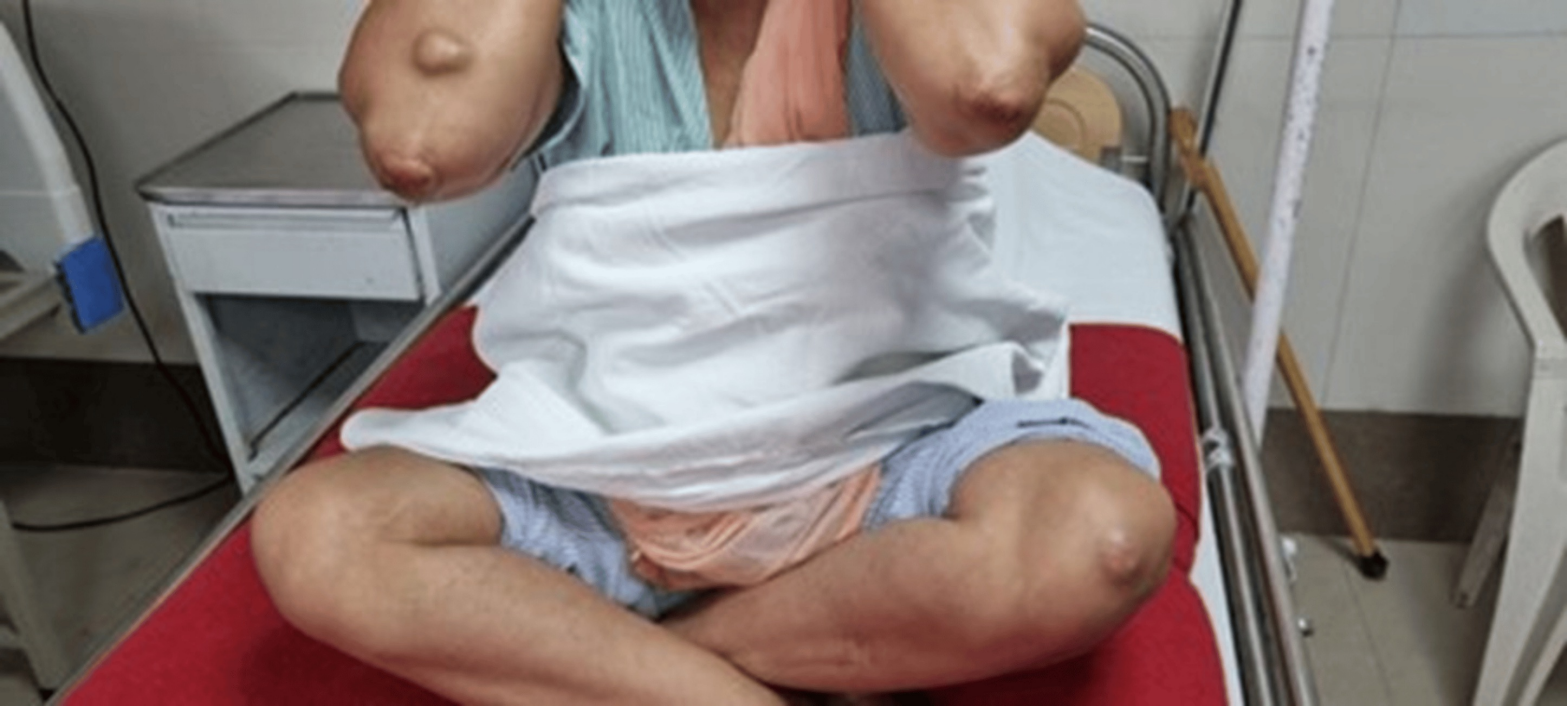
Xanthomas over the bilateral elbows and left knee

**Figure 4:**
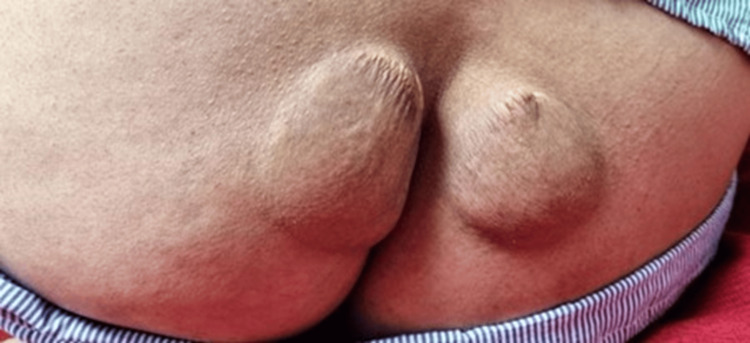
Xanthomas over the buttocks

**Figure 5:**
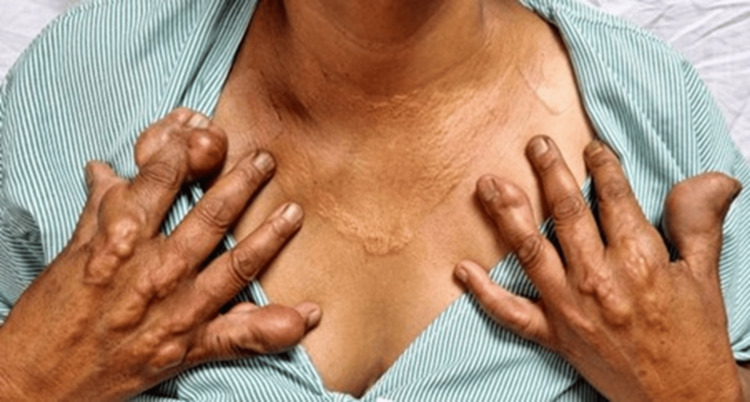
Xanthomatous lesion on the upper chest and neck

A 40-year-old unmarried woman presented with worsening exertional anginal symptoms for the past six months. She was non-obese and a non-smoker and had no history of diabetes mellitus or hypertension. On initial presentation, the patient's vital signs were as follows: heart rate 76 beats per minute, blood pressure 134/86 mmHg (right arm) and 132/84 mmHg (left arm), respiratory rate 16 per minute, temperature 36.8°C, and body mass index (BMI) 23.7 kg/m² (weight 62 kg; height 162 cm). No significant difference was noted between bilateral arm blood pressures. On examination, she exhibited multiple, firm, non-tender xanthomas over the dorsal aspect of both hands (~2.5-3 cm diameter) (Figure 1) and feet (Figure 2) and the extensor aspect of elbows and knees (~2 cm) (Figure 3) and buttocks (Figure 4) and a confluent lesion over the neck and upper chest (Figure 5), consistent with both tuberous and tendon xanthomas. There were no Achilles tendon swelling/tenderness and no evidence of palmar xanthomas.

**Figure 6:**
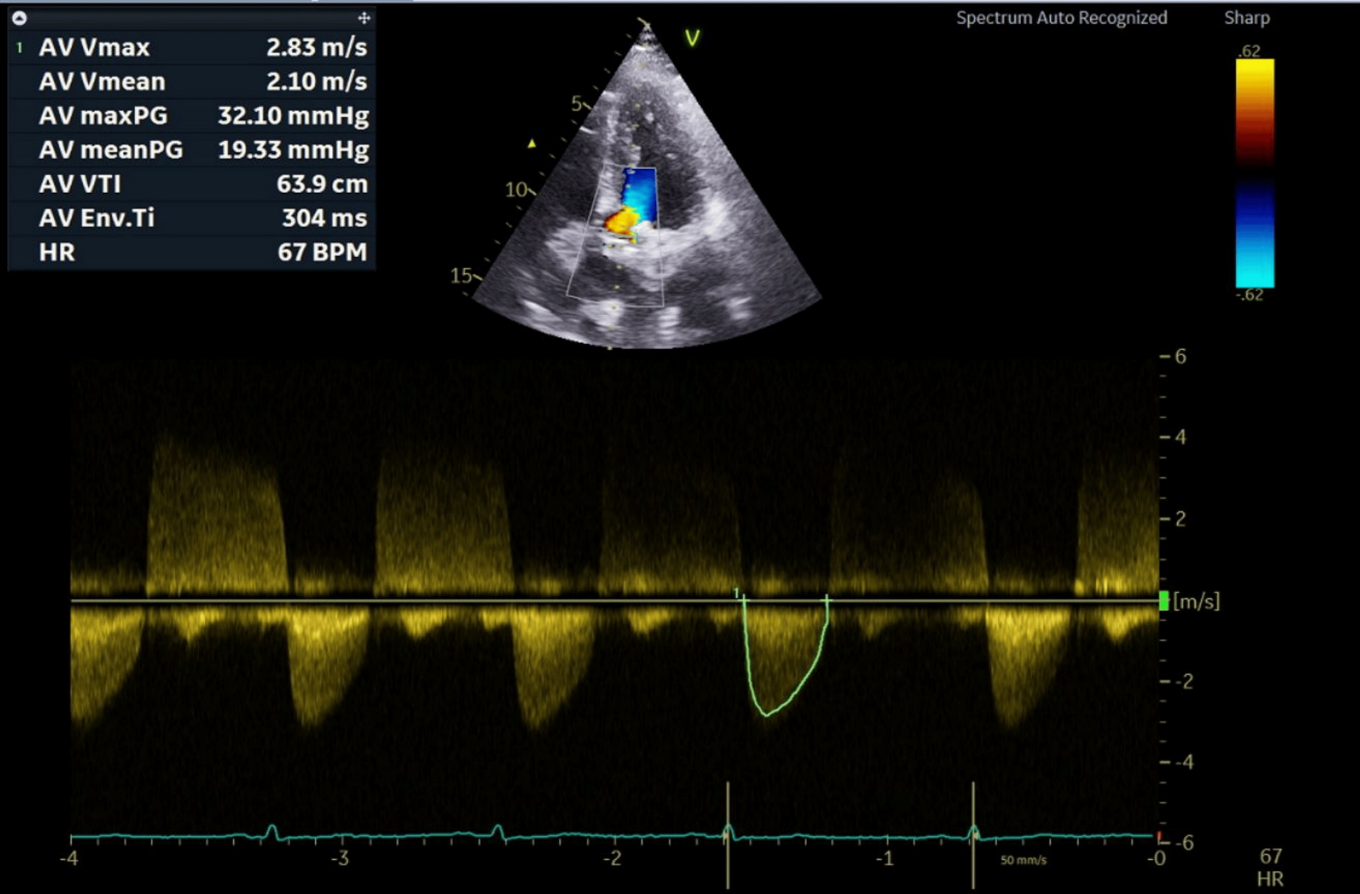
Echocardiogram, apical five-chamber view, showing mild aortic stenosis and mild aortic regurgitation

On cardiac auscultation, a soft systolic murmur was heard over the aortic area, and a left carotid bruit was heard. She explained that she developed these skin lesions at the age of 25 but never underwent any evaluation. Her family history was unremarkable, except that her father died at the age of 43 due to a myocardial infarction. He had no xanthoma or xanthelasma. Neither her mother nor her siblings had any history of cardiovascular events or any form of skin manifestations of FH. Her lipid profile revealed a total cholesterol level of 485 mg/dl, an LDL-C level of 398 mg/dl, a high-density lipoprotein cholesterol (HDL-C) level of 46 mg/dl, and a triglyceride level of 110 mg/dl. Other laboratory tests, including hemogram, renal function, liver function, thyroid function, and urine examination, were normal. Her electrocardiogram findings were normal. Echocardiography revealed a mildly thickened aortic valve with a mean gradient of 20 mmHg and a peak gradient of 32 mmHg, compatible with mild aortic stenosis. Mild aortic regurgitation was noted with a regurgitant fraction of approximately 20%. No regional wall motion abnormality was present; LVEF was preserved at 64% (Figure 6).

**Figure 7:**
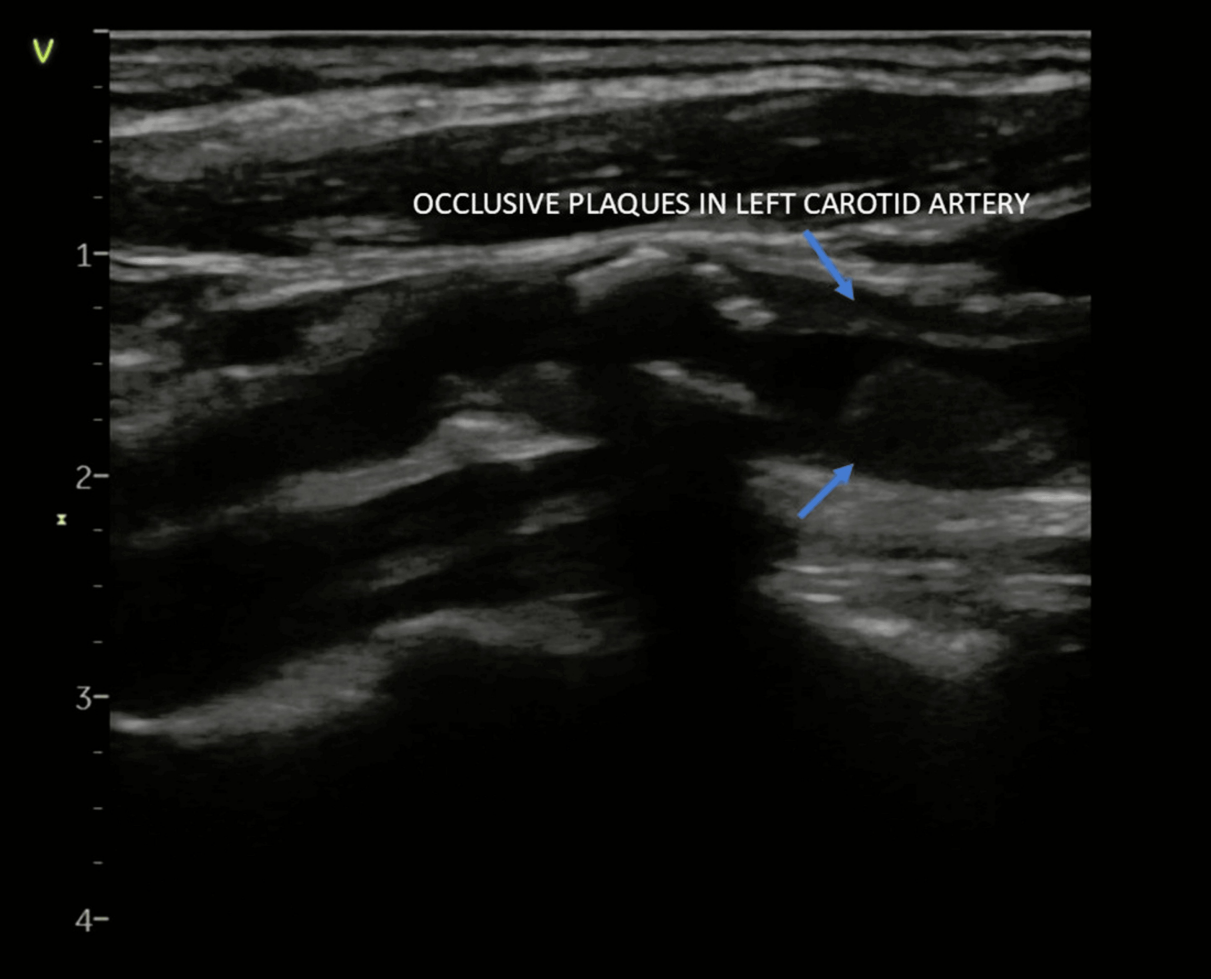
Duplex scan image showing significant stenosis in the left carotid artery

Duplex ultrasound revealed significant narrowing of the left and right common carotid arteries, 70% stenosis of the left internal carotid artery (peak systolic velocity: 238 cm/s), and 65% stenosis of the right internal carotid artery (peak systolic velocity: 212 cm/s) (Figure 7).

**Figure 8:**
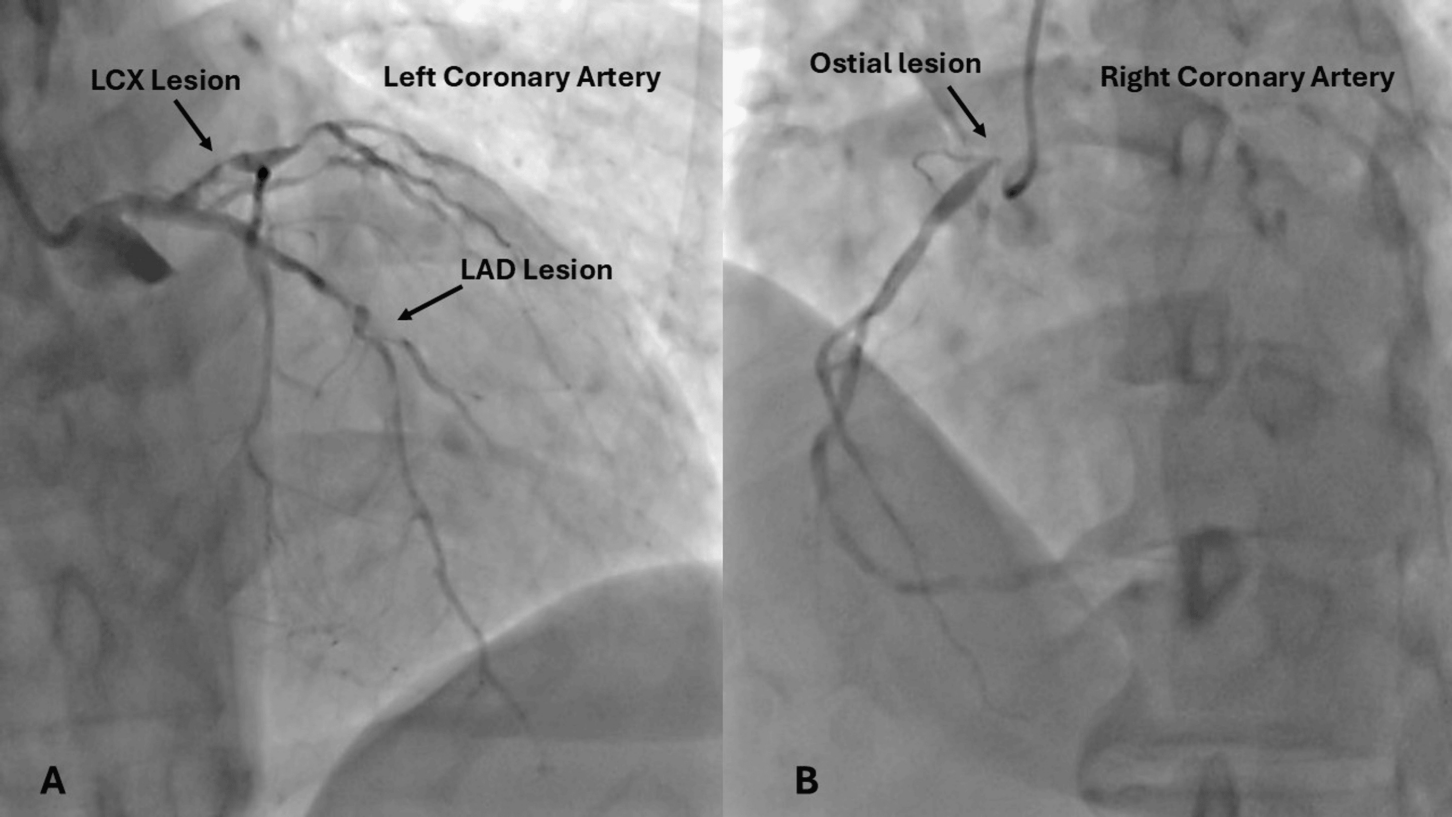
Diffuse disease in both left and right coronary arteries. Note the critical stenosis in LAD and LCX coronary arteries as marked with arrows (A) and critical ostial stenosis in the right coronary artery as marked with an arrow (B) LAD: left anterior descending; LCX: left circumflex

Coronary angiography revealed aortic root calcification (Video 1) and critical diffuse triple-vessel disease characterized by a 90% mid-segment stenosis in the left anterior descending artery, a 90% proximal stenosis in the left circumflex artery, and a 95% ostial stenosis in the right coronary artery. Extensive multivessel disease and a high level of anatomic complexity guided the recommendation for surgical revascularization (Figure 8, Video 2, and Video 3).

**Video 1:** Aortic root calcification

**Video 2:** Left coronary angiogram showing diffuse disease in the left coronary artery

**Video 3:** Right coronary artery angiography

The physical examination, lipid profile, and family history of premature CAD in the father were compatible with the diagnosis of FH. Although genetic analysis is the gold standard for confirmation, it was not performed in this case due to financial constraints and patient refusal. She was advised to undergo coronary artery bypass grafting along with optimal medical therapy for angina relief. For the treatment of high LDL-C, she was prescribed 40 mg of rosuvastatin and 10 mg of ezetimibe. She was also advised to take PCSK9 inhibitors. However, she was not willing either to undergo surgery or to take PCSK9 inhibitors, primarily because of financial constraints, and was later lost to follow-up.

## Discussion

FH encompasses a spectrum of inherited disorders leading to impaired clearance of LDL-C, resulting in lifelong, markedly elevated cholesterol and dramatically increased risk of premature CAD [4-8]. The differential diagnosis of FH includes heterozygous FH, which typically presents with LDL-C levels above 190 mg/dl and premature ASCVD, as well as homozygous FH, which manifests with even higher LDL-C (>500 mg/dl untreated), more extensive xanthomas, and aortic valve disease, indicative of angiographically advanced CAD at a much younger age [5]. Autosomal recessive hypercholesterolemia (ARH) should be considered in cases lacking a dominant inheritance pattern [4-6]. This case, showing florid cutaneous xanthomas and diffuse severe coronary/valvular/vascular involvement, represents the classical clinical spectrum of FH, though genetic confirmation was not available due to resource constraints. Recent studies highlight the critical contribution of elevated lipoprotein(a) (Lp(a)) to cardiovascular risk in FH [7]. Lp(a) is an independent, genetically determined proatherogenic particle, frequently elevated in FH patients, and should be measured routinely, as levels above 50 mg/dl further increase risk and guide intensified management. Lp(a) also confounds LDL-C assays and may be present in phenotypic FH without detectable monogenic variants [7].

Globally, FH prevalence varies, but contemporary guideline statements estimate up to one in 250 adults may be affected, with higher rates noted in South Asian populations and among those presenting with premature CAD [8,9]. Despite this, over 90% of cases remain undiagnosed due to gaps in physician awareness, lack of cascade family screening, and resource limitations. Optimal management involves achieving LDL-C targets below 70 mg/dl (or <55 mg/dl for those with recurrent ASCVD), yet global registry data demonstrate most patients are far from target [8,9].

The pathophysiology of FH involves defective LDL receptor-mediated endocytosis resulting in the accumulation of LDL-C and sometimes Lp(a) in plasma, the initiation of early arterial intimal plaque formation, endothelial dysfunction, and accelerated atherosclerosis [4,7,8]. The nature (null vs. defective) and location of the mutation dictate clinical severity, with compound or double heterozygotes often displaying phenotypes approaching homozygous FH [7]. The cardiovascular manifestations include not only premature and often multivessel CAD but also carotid and peripheral artery disease, aortic valve stenosis and regurgitation, and generalized xanthomatous deposits as seen in this report [1,7].

Genetic testing, while the gold standard for FH diagnosis and critical for cascade screening, faces significant logistical, cost, and access barriers in many regions, often necessitating a clinical diagnosis based on criteria such as the Simon Broome or Dutch Lipid Clinic Network. Importantly, the absence of genetic confirmation should never delay or compromise aggressive risk factor modification [7,9].

Pharmacological management has evolved with the introduction of new agents. For heterozygous FH, high-intensity statins are recommended along with other drugs such as ezetimibe and bile acid sequestrants. PCSK9 inhibitors and inclisiran are strongly recommended to further reduce LDL-C levels. Apheresis is indicated in patients with progressive ASCVD at very high risk. In patients with homozygous FH, the mainstays of therapy include PCSK9 inhibitors, lomitapide, mipomersen, apheresis, and liver transplant [8-10]. Unfortunately, besides statins and ezetimibe, the remaining treatment modalities are not widely available and are out of reach, especially for poor patients, as we commonly encounter in India. Inclisiran, a small interfering RNA targeting hepatic PCSK9 synthesis, offers twice-yearly dosing and robust LDL-C reduction. The most recent meta-analysis confirms inclisiran's safety profile, demonstrating rates of adverse events comparable to control with no increase in major cardiovascular or hepatic complications and significant sustained LDL-C lowering in both primary and secondary prevention, including FH populations [10]. Although cost and local access remain hurdles in many parts of India, inclisiran represents a promising tool to address persistent LDL-C elevation, particularly in patients unable to access other injectable therapies or with adherence barriers to more frequent regimens.

This case is unique for its classic clinical presentation of massive cutaneous and tendinous xanthomas, triple-vessel critical CAD, and extracoronary aortic and carotid involvement in a young Indian woman. It is among the very few reports from this region to document comprehensive multimodal vascular imaging (including carotid duplex) and discuss therapeutic options, highlighting the current gaps in diagnosis, genetic confirmation, and access to advanced therapies such as PCSK9 inhibitors and inclisiran. The findings add to world literature by substantiating severe, diffuse ASCVD phenotypes in Indian FH patients and illustrate the urgent need for system-wide screening, better risk stratification (including Lp(a)), and equitable access to modern lipid-lowering options.

## Conclusions

FH remains underdiagnosed and undertreated globally, especially among South Asian populations at heightened risk for early ASCVD. This case exemplifies the entire clinical and imaging spectrum of FH, from massive skin and tendon xanthomas to premature, complex coronary and extracoronary vascular disease, reinforcing the importance of comprehensive triage, early Lp(a) testing, and aggressive LDL-C lowering. With advances such as inclisiran, there is new hope for durable, accessible cholesterol management, but achieving optimal outcomes will require widespread physician education, improved genetic and biochemical diagnostic access, and system-wide efforts for cascade screening and therapy. Improved recognition and individualized care can fundamentally alter the natural history of FH and curb the devastating burden of cardiovascular disease in high-risk populations.
